# Novel advances in monitoring and therapeutic approaches in idiopathic intracranial hypertension

**DOI:** 10.1097/WCO.0000000000000690

**Published:** 2019-03-06

**Authors:** James L. Mitchell, Susan P. Mollan, Vivek Vijay, Alexandra J. Sinclair

**Affiliations:** a Metabolic Neurology, Institute of Metabolism and Systems Research, University of Birmingham; b Department of Neurology, University Hospitals Birmingham; c Birmingham Neuro-Ophthalmology, Queen Elizabeth Hospital; d Centre for Endocrinology, Diabetes and Metabolism, Birmingham Health Partners, Birmingham, UK

**Keywords:** glucagon-like peptide 1 receptor agonists, idiopathic intracranial hypertension, intracranial telemetry, novel therapies, optical coherence tomography, raised intracranial pressure

## Abstract

**Purpose of review:**

The current article appraises the recent developments in idiopathic intracranial hypertension (IIH), with particular attention to novel therapeutic avenues and advanced clinical assessment and monitoring with optical coherence tomography and telemetric intracranial pressure devices.

**Recent findings:**

The incidence of IIH is increasing. The first consensus guidelines for IIH have been published detailing investigation and management algorithms for adult IIH. Improved understanding, clinical assessment and monitoring are emerging with the use of optical coherence tomography. Intracranial pressure telemetry is providing unique insights into the physiology of raised intracranial pressure in IIH. There are now an increasing number of ongoing clinical trials evaluating weight loss methods and novel targeted therapies, such as 11ß-HSD1 inhibition and Glucagon-like peptide 1 (GLP-1) receptor agonists.

**Summary:**

Several studies are evaluating new therapies for IIH. Monitoring techniques are advancing, aiding diagnosis and allowing the clinician to accurately evaluate changes in papilloedema and intracranial pressure.

## INTRODUCTION

Idiopathic intracranial hypertension (IIH) is characterized by increased intracranial pressure (ICP) with no identifiable cause. IIH, also known as pseudotumor cerebri, is a syndrome with the major risk factor of recent weight gain, occurring mainly in overweight women of working age [1^▪▪^,^2^]. There is a rising incidence in this disease [3] and the incidence appears related to country-specific prevalence of obesity [4].

In the majority of those presenting with IIH, they will have headache that is progressively more severe and frequent, with a divergence of traditional considerations of a raised ICP headache [5] to a phenotype that is highly variable and commonly mimics migraine [6,7^▪^]. Other reported symptoms include transient visual obscurations (unilateral or bilateral darkening of the vision typically lasting seconds), pulsatile tinnitus, back pain, dizziness, neck pain, visual blurring, cognitive disturbances, radicular pain, and horizontal diplopia [2,8^▪^,^9^]. Prognosis is variable as IIH can either be self-limited or have a lifelong chronic course with significant affects on quality of life [10,11].

In 2018, the first consensus IIH guidance was published [1^▪▪^]. The document was reviewed by a committee of international key opinion leaders and a patient group, which established a James Lind Alliance Priority Setting Partnership for adult IIH [12]. It sets out key diagnostic and management principles. The diagnostic principles of the investigation of papilloedema are to find any underlying treatable cause in a timely manner, protect the vision and ensure timely re-examination when vision is at risk, and to enable onward care of the patient with the input from the most appropriate experienced clinician. Key considerations are to exclude secondary causes, such as venous sinus thrombosis with appropriate imaging and check blood pressure to exclude malignant hypertension. The Friedman *et al.*[13] 2013 diagnostic criteria are used, although a key area of uncertainty still exists with the diagnostic cut-off, lumbar puncture opening pressure (LP OP) 25 cm cerebrospinal fluid (CSF) as was then recognized. A grey zone between 25 and 30 cm CSF exists with the recommendation that wherever measured LP OP does not fit the clinical picture, consideration should be given to repeat measurement or ICP monitoring.

The key management principles are addressing the underlying modifiable risk factor of weight gain; protecting the vision through regular assessment and escalation of treatment when sight is threatened; and reducing headache morbidity through active management. Importantly, considerations included the indication for CSF diversion surgery in declining visual function. However, alternative interventions, such as neurovascular stenting do not yet have evidence to recommend them. It should also be stressed that headache alone is not an indication for CSF diversion with a majority of patients having persistent headache following the procedure [14].

The major achievement of this document is the interdisciplinary working to provide a framework to standardize care for those with IIH. This standardized approach to care has been subsequently published in the European Headache Federation Guidelines for IIH [15]. 

**Box 1:**
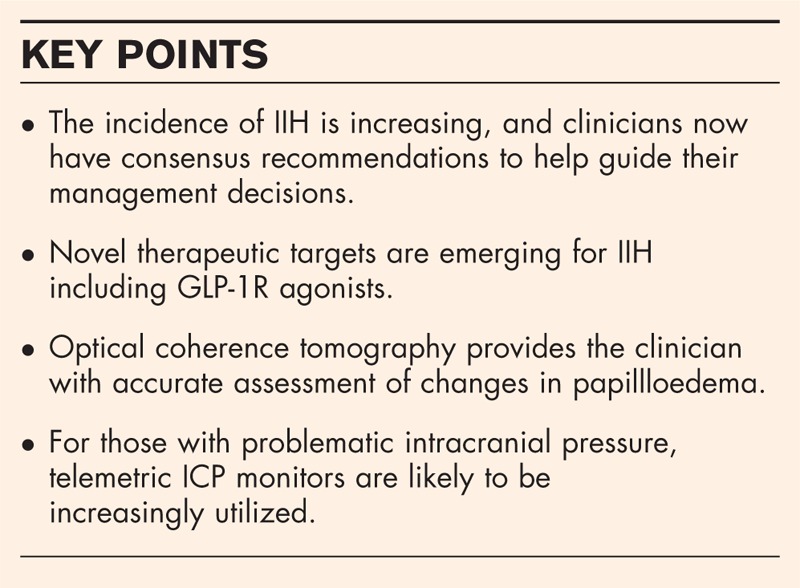
no caption available

## ADVANCES IN OCULAR IMAGING

Visual monitoring of patients is a key principle of management [1^▪▪^], in addition to visual field perimetry, optical coherence tomography (OCT) has allowed new observations in papilloedema. OCT is a rapid, reliable, reproducible and noninvasive imaging technique, using reflected light waves to produce high-resolution cross-sectional and 3D representations of retinal structures. Optic nerve head (ONH) OCT measures have been correlated with the modified Frisén grading of papilloedema [16,17]. The noninvasive nature of these techniques make them ideal in follow-up in contrast to lumbar puncture, which is feared by patients [18].

**FIGURE 1:**
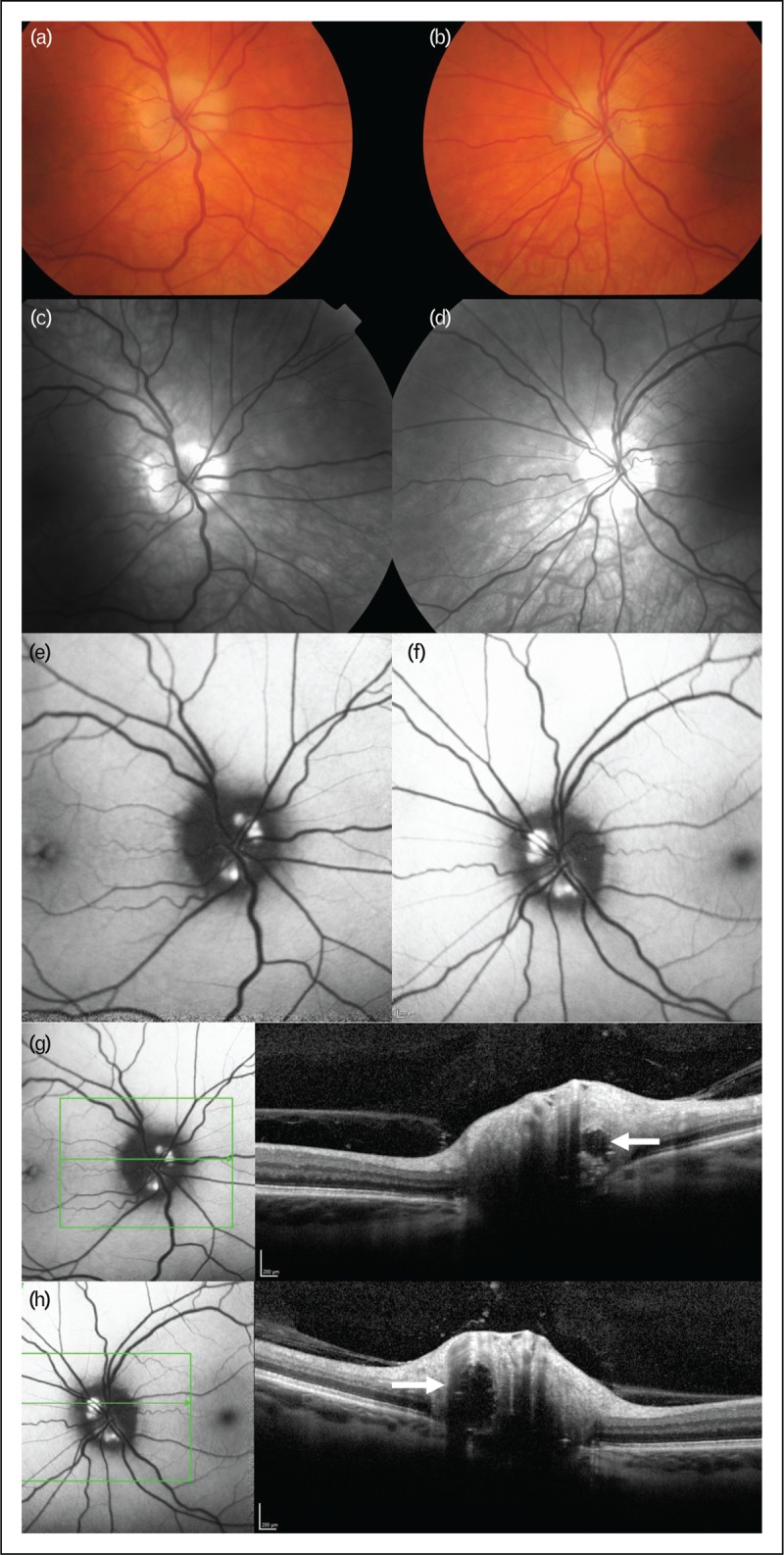
(a) Fundus photograph of the right optic disc in a patient referred for papilloedema. The optic nerve shape is irregular. Note there is no loss of view of any of the retinal vessels as they run over the border of the optic nerve. (b) Fundus photograph of the left optic disc in a patient referred for papilloedema. (c) Red-free fundus photograph of the right optic disc highlights hyper reflectivity at the optic nerve head. (d) Red-free fundus photograph of the left optic disc highlights hyper reflectivity at the optic nerve head. (e) Blue autoflurorescence (BAF) imaging using the Heidelberg Spectralis optic coherence tomography (OCT) imaging. This clearly highlights buried optic disc drusen as a cause of the pseudopapilloedema. (f) BAF OCT imaging clearly highlights buried optic disc drusen in the left eye as a cause of the pseudopapilloedema. (g) BAF and an OCT disc volume imaging cross-section showing one of the drusen (white arrow) in the right eye and the depth of the drusen with obvious elevation of the over laying optic nerve tissue. (h) BAF and an OCT disc volume imaging cross-section showing one of the drusen in the left eye (white arrow) and the depth of the drusen with obvious elevation of the over laying optic nerve tissue.

**FIGURE 2:**
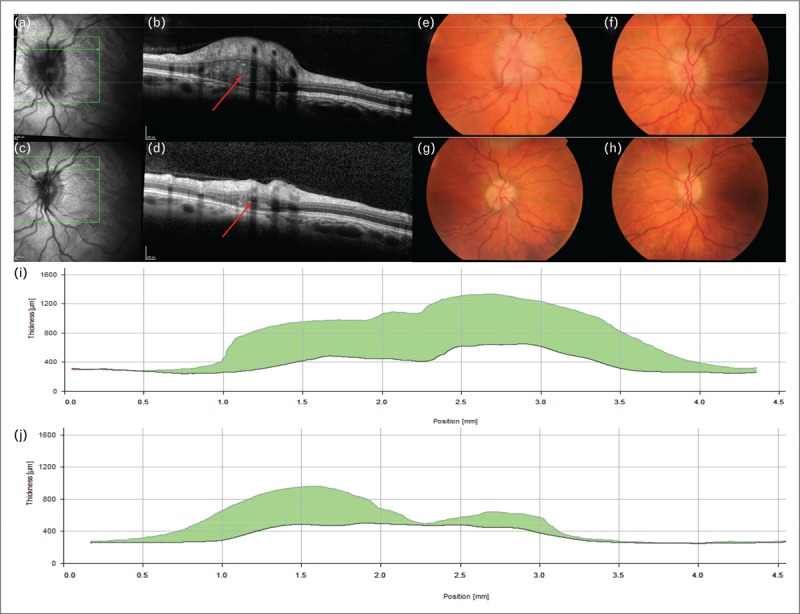
(a) Optical coherence tomography infra-red image, in same patient as other figures, shows the cross-sectional cut for (b) with the arrowed line. (b) OCT cross-sectional volume image shows a typical peripapilliary hyperreflective ovoid mass-like structures (PHOMS) (arrow). (c) OCT infra-red image, in same patient as other figures, shows the cross-sectional cut for (S) with the arrowed line. Note the reduction in optic nerve head swelling. (d) OCT cross-sectional volume image shows the reduction in the size of the PHOMS (arrow). (e and f) Fundus photographs of the right (e) and left (f) eye at baseline showing Frisen grade 4 papilloedema. (g and h) Resolution of papilloedema following bariatric surgery seen on fundus photographs. (i and j) Resolution of papilloedema seen on OCT as reduction of total retinal thickness at the optic nerve head in right (i) and left (j) eyes. OCT, optical coherence tomography.

Whenever investigating papilloedema, OCT is useful in the differentiation of pseudopapilloedema from true papilloedema, a key area of misdiagnosis [19,20]. Combining blue autofluorescence and disc volume OCT scanning can highlight buried crystalline drusen clearly (Fig. 1). There is debate regarding OHN drusen that appear de novo in papilloedema. Peripapillary hyperreflective mass-like structures, termed PHOMS (Fig. 2), [21,22] may be nerve fibre in origin [21]. Further work may define their significance.

Standard measurements for papilloedema include peripapillary retinal nerve fibre layer (pRNFL) and ONH volume. These are reliably increased in active IIH compared with controls, are significantly associated with CSF opening pressure and improve following treatment [23]. OCT has revealed dynamic deformation of the peripapillary retinal pigment epithelium and Bruch's membrane (pRPE/BM) regressing towards the normal shape with reduction of ICP [24,25]. Deformation in pRPE/BM may be of particular value in evaluating atrophic papilloedema with minimal RNFL swelling, as deflection of pRPE/BM may correlate with disease activity. Macular RNFL thickness has been shown to be significantly reduced compared with controls, reduces over time and is associated with ONH volume measurements at baseline and visual function [26,27].

OCT angiography (OCTA) is a relatively new, noninvasive investigative modality, allowing visualization of ONH vasculature. OCTA is based on detecting differences in amplitude, intensity or phase variance between sequential B-scans taken at the same location of the retina. Early use has been directed at papilloedema is with small cohorts, reported on multiple OCT platforms, with differing methodology. The advantage of OCTA is the ability to segment layers and view below retinal haemorrhage, but despite the small field of view, there is high resolution [28^▪^]. Early findings include dilated tortuous OHN capillaries with no vascular dropout in comparison to ischaemic OHN oedema where there is vascular dropout [29]. OCTA may provide useful biomarkers and aide diagnosis as fundus fluorescence angiography, its predecessor, remains an invasive test [30^▪^].

OCT technology is limited by penetration particularly in severe OHN swelling. Measurement of the pRPE/BM deflection is semi-automated and accurately placing landmarks is challenging [25]. Proprietary automated algorithms commonly fail to correctly segment the layers, and manual re-segmentation is recommended [15,31]. Overall, as the different parameters of OCT are investigated, the outstanding advantage of OCT is the provision of reliable quantitative method for the longitudinal monitoring of IIH patients (Fig. 2).

## ADVANCES IN INTRACRANIAL PRESSURE TELEMETRY

The most common method of ICP measurement in IIH remains lumbar puncture, with several well documented negative aspects [32]. Direct measurement of ICP is either noninvasive (yet to be used routinely) or invasive. Invasive ICP measurement can be performed at various anatomical sites namely intraventricular, intraparenchymal, subarachnoid, subdural, epidural; where there is CSF communication ICP can be measured by lumbar puncture [33]. Basic ICP measurement by external ventricular, lumbar drains or lumbar puncture is made by fluid column; more recently a variety of microsensors have become available that locate in the target tissue. These have been limited by drift phenomena and the requirement for external wiring, thus the risk of infection with use in excess of 72 h.

Telemetric ICP monitors are now available commercially. There are two main systems, Neurovent p-Tel, Raumedic, Helmbrechts, Germany and Sensor Reservoir, Miethke, Potsdam, Germany. The wiring issue is resolved with wireless power and reading utilizing induction technology. The drift issue is solved in both systems by way of an external monitor reading atmospheric pressure and solid-state sensor technology. The two systems differ with Neurovent p-Tel siting a pressure sensor in the brain parenchyma; meanwhile the Miethke system is based on a sensor within a reservoir attached to a ventricular drain. Both devices are readable by external hand-held equipment, both devices are passive in the sense that they have no power or memory integral to the device and so pressure is only recorded with the external equipment *in situ*.

The major initial application for the Miethke system is in refining valve settings in challenging patients, a recent case series highlights this application, of note the highest frequency of valve adjustments was seen in the IIH cohort [34].

**FIGURE 3:**
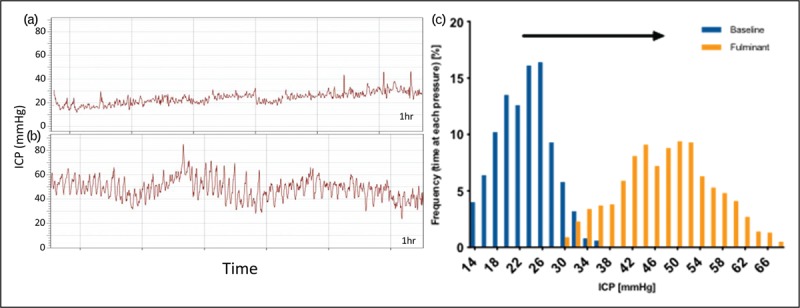
(a) Intracranial pressure telemetry, 1 h baseline recording of patient with intracranial hypertension. Mean 23.8 mmHg (32.3 cm CSF), range 11.8–46.5 mmHg. (b) Above patient during presentation with fulminant IIH. Mean 48.6 mmHg (66.1 cm CSF) range 23.6–85.0 mmHg. Note peak values of 85 mmHg (115.6 cm CSF). (c) Histogram of pressure recordings from (a) and (b) – arrow demonstrates right shift with increasing pressure and waveform variability. CSF, cerebrospinal fluid.

More data is available for the Raumedic p-Tel as it has been available for longer. It sites a solid-state pressure sensor approximately 20 mm into the brain parenchyma, usually within the right frontal lobe. From the largest published series, there is a low complication rate, approximately 6% overall, with seizures affecting 3% and infection in 1.5% [35^▪▪^]; however, this was in a series of patients with significant structural brain abnormality (for example, hydrocephalus) and the rate of such complications is likely lower in IIH patients. Of note, the UK driving regulations allow resumption of driving 1 week after insertion in patients without complications. The device provides a high degree of accuracy with low drift [36]. Many have been kept *in situ* beyond the licensed 3-month period [35^▪▪^,^37^]; where they have been shown to retain their accuracy with low drift of 2.5 mmHg over a median 241-day implantation period. The device samples at 5 Hz, considerably lower than the wired and Miethke systems, although this is sufficient for waveform analysis [36,38]. The device is capable of long-term recordings for up to 1 week with the present hardware and can be worn by an ambulant patient out with the hospital environment (Fig. 3) [38].

Telemetric ICP monitors have an evolving role in diagnosis and monitoring of several conditions. In IIH, particular roles could include evaluating whether neurosurgical shunt placement is advised in a deteriorating patient developing fulminant disease. Furthermore, it is useful in evaluating whether pressure is pathologically elevated in those with minimal ocular features and in shunted patients. Monitors can inform the setting of CSF shunt valves aiming to abrogate low pressure headaches, at present seen in 23% [14]. ICP telemetry may also facilitate the differentiation between raised pressure headaches and migrainous headaches [39].

## NOVEL THERAPEUTICS AND INTRACRANIAL HYPERTENSION

**FIGURE 4:**
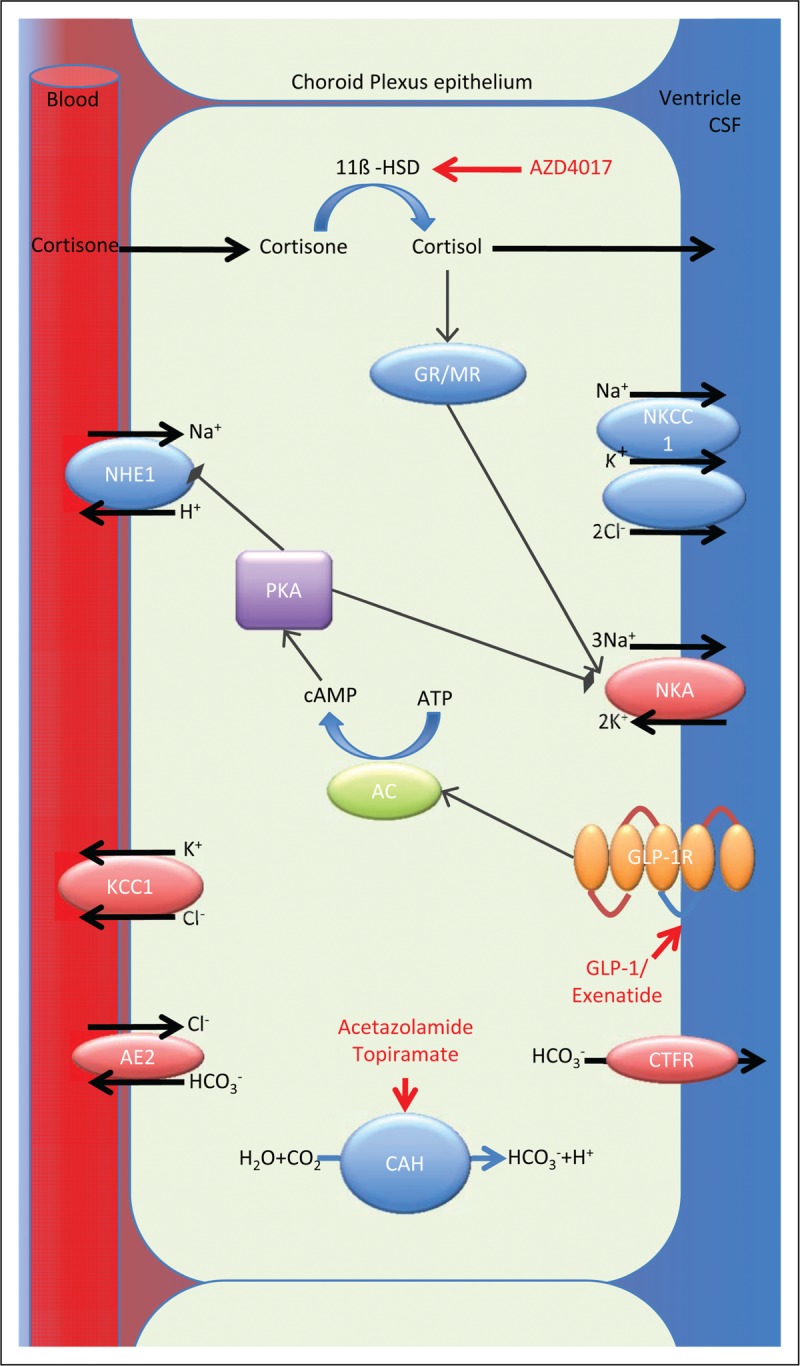
The major ion channels responsible for CSF secretion in the choroid plexus are shown with sites of action of acetazolamide, AZD4017 and exenatide. Cortisone is converted to the active cortisol by 11ß-HSD1, cortisol binds to the GR and MR receptors, which upregulate Na^+^ K^+^ ATPase activity; AZD4017 inhibits 11ß-HSD1 reducing local availability of cortisol. Exenatide binds and activates GLP-1R stimulating the conversion of ATP to cAMP by AC. cAMP activates PKA, which inhibits the Na^+^ H^+^ exchanger reducing Na^+^ re-absorption and also inhibits the Na^+^ K^+^ ATPase reducing Na^+^ excretion. Carbonic anhydrase catalyzes the conversion of H_2_O and CO_2_ to H^+^ and HCO_3_^−^, which is important in the establishment of the osmotic gradient. Both acetazolamide and topiramate inhibit carbonic anhydrase function. AC, adenylate cyclase; AE2, anion exchange protein 2; cAMP, cyclic adenosine monophosphate; CSF, cerebrospinal fluid; CTFR, cystic fibrosis transmembrane conductance regulator; GLP-1: glucagon-like peptide 1; GLP-1R: glucagon-like peptide 1 receptor; 11ß-HSD, 11ß-hydroxysteroid dehydrogenase type 1; GR/MR, glucocorticoid and mineralocorticoid receptors; KCC1, K-Cl cotransporter 1; NHE1, Na-H antiporter; NKA, N-K ATPase; NKCC1, Na-K-Cl cotransporter; PKA, protein kinase A.

Acetazolamide is the longest established treatment for IIH. In 2015, following the publication of the first two randomized control trials for medical treatment in IIH [8^▪^,^40^], an updated Cochrane review highlighted that there was insufficient evidence to recommend or reject the efficacy of acetazolamide for treating IIH and insufficient evidence for other drugs used in IIH [41]. Of note, there was no effect of acetazolamide on headache seen in the IIHTT [6]. The common existing drugs used in IIH have been evaluated acutely *in vivo* at clinically relevant doses, and were not found to significantly reduce ICP, with the exception of topiramate [42^▪^]. There is, therefore, an unmet need for novel therapeutic strategies in IIH (Fig. 4).

Disordered CSF dynamics have been suspected to underlie the raised ICP seen in IIH. There are currently no novel drugs targeting the underlying pathogenesis driving IIH, which remains elusive. Novel therapies are currently focussed on reducing ICP through reducing CSF secretion. Ideally novel therapies would also reduce weight as this approach is disease modifying in IIH [43^▪▪^].

The choroid plexus is the principle site of CSF production; this is driven by the net movement of sodium ions (Na^+^) from the blood to the cerebral ventricles, creating an osmotic gradient down, which water moves. Although several channels are involved in this process, the principle channel is the Na^+^ and K^+^-dependent adenosine triphosphatase (Na^+^/K^+^/ATPase) that actively transports Na^+^ into the cerebral ventricle and is the rate-limiting step [44,45].

## 11ß-HYDROXYSTEROID DEHYDROGENASE TYPE 1

11ß-hydroxysteroid dehydrogenase type 1 (11ß-HSD1) is an intracellular enzyme that converts inactive cortisone to the active cortisol. This amplifies local glucocorticoid activity independent of systemic cortisol. 11ß-HSD1 expression and activity has been demonstrated in choroid plexus epithelial cells, along with other key elements of the glucocorticoid signalling pathway [46,47]. Inhibitors have been developed, including AZD4017, originally as potential therapies for diabetes mellitus type 2 and the metabolic syndrome. Glucocorticoid metabolism has been characterized in IIH in relation to therapeutic weight reduction; global 11ß-HSD1 activity decreased with weight loss as measured by urinary glucocorticoid metabolites by gas chromatography/mass spectroscopy [43^▪▪^]. Importantly it was noted that the reduction in ICP significantly correlated with reduction in 11ß-HSD1 activity [47]. Of interest, is that 11ß-HSD1 inhibition reduced intraocular pressure and it has been shown that secretory mechanisms of the ocular ciliary body are akin to that of choroid plexus epithelium [46–48].

11ß-HSD1 inhibitors do not affect systemic glucocorticoid metabolism [49], but would reduce CSF secretion though reducing local cortisol availability in the choroid plexus with subsequent reduction of downstream glucocorticoid receptor-mediated sodium transportation, reduced osmotic gradient and decreased water movement into the cerebral ventricle [46,47]. Conversely systemic administration of glucocorticoids has been found to precipitate intracranial hypertension [50]. The IIH Drug trial (IIHDT), clinicaltrials.gov identifier NCT02017444, has investigated the ability of an 11ß-HSD1 inhibitor to reduce CSF secretion and hence ICP in patients with IIH [51^▪^]. IIHDT is the first phase 2 double-blind placebo-controlled trial in IIH. It recently completed recruitment and is expected to report in 2019.

## GLUCAGON-LIKE PEPTIDE 1

The incretin glucagon-like peptide 1 (GLP-1) is a gut peptide secreted by the distal small intestine in response to food intake [52]. GLP-1 stimulates glucose-dependant insulin secretion and inhibits glucagon release, lowering blood glucose only in the presence of insulin and not resulting in hypoglycaemia [53]. GLP-1 is also synthesized in neurons of the nucleus tractus solaris that project to the hypothalamus [54] and promotes satiety and weight loss [55]. GLP-1 analogues have a clinical role in the management of type 2 diabetes mellitus, as well as for weight loss in obesity. Several GLP-1 agonists have been developed and are now licensed drugs [56]. These include exenatide twice daily, exenatide once weekly, liraglutide, lixisenatide, albiglutide, dulaglutide and most recently semaglutide. Currently, only liraglutide is licensed for weight loss in obesity. They vary in structure and pharmacology, ability to penetrate the blood–brain barrier (BBB) as evidenced by CNS effects. Importantly, the choroid plexus epithelium lies outside the BBB [57].

GLP-1 also has a diuretic effect by reducing Na^+^ re-absorption in the renal proximal tubule, thereby increasing Na^+^ and water excretion [58]. Activation of GLP-1R stimulates the conversion of adenosine triphosphate to cyclic adenosine monophosphate (cAMP) by adenylate cyclase. cAMP activates protein kinase A, which inhibits the Na^+^ H^+^ exchanger, thus reducing Na^+^ re-absorption. Choroid plexus epithelial cell function is inverted compared with renal proximal tubule but with an analogous fluid transport mechanism [59], and as such GLP-1R was investigated as a potential target for conditions with raised ICP. It has been shown that GLP-1 receptor (GLP-1R) is expressed in the human choroid plexus. Treatment with the agonist exendin-4 modulates the GLP-1R in the rat choroid plexus through agonist induced receptor internalization, which was shown to increase cAMP generation and reduce Na^+^/K^+^/ATPase activity. Importantly exendin-4 reduced ICP in conscious rats at clinically relevant doses. There was a 65% reduction in ICP 30 min post dose compared with baseline and a cumulative effect seen with reduction in the ICP to 79.3 and 72.5% of baseline values predose at days 2 and 4, respectively, of the experiment. The action was blocked by intraventricular administration of the GLP-1R antagonist exendin 9-39, suggesting the effect is mediated by the GLP-1R in the brain. Importantly, the effect was also seen in a rat model with markedly raised ICP [60^▪▪^]. The IIH Pressure Trial, ISRCTN12678718, is a double-blinded, placebo-controlled physiology study assessing the effects of exenatide, a GLP-1R agonist, on ICP in a cohort with active IIH and is expected to report this year [39].

There are also developments with surgical management of IIH, two trials are currently ongoing, the IIH Weight Trial [61] (ClinicalTrials.gov Identifier: NCT02124486) is a randomized controlled trial of Bariatric Surgery Versus a Community Weight Loss Programme and opened to recruitment in 2014. The SIGHT trial (ClinicalTrials.gov Identifier: NCT03501966) opened in 2018 and is a triple-arm randomized controlled trial of medical therapy (acetazolamide) vs. medical therapy with Optic Nerve Sheath Fenestration vs. medical therapy with ventriculoperitoneal shunting.

## CONCLUSION

The incidence of IIH is increasing along with global rates of obesity, a key pathological factor, making IIH research and management increasingly important. The first consensus guidelines for the management of IIH have now been published guiding the clinical management of this condition. Looking to the future, there are several new avenues of clinical therapeutics based on reducing CSF secretion, with GLP-1 receptor agonists appearing promising as they also significantly reduce weight. Advances in OCT technology will continue to improve our diagnosis and monitoring of papilloedema, and may provide unique biomarkers. Early indications show that telemetric ICP monitoring may provide insight into CSF dynamics in IIH and facilitate management decisions.

## Acknowledgements

Contributor Statement: All authors have read and approved the final manuscript.

### Financial support and sponsorship

None.

### Conflicts of interest

A.J.S. is an inventor and the University of Birmingham applicant on patent application no. PCT/GB2015/052453 related to this work entitled ‘Elevated intracranial pressure treatment.’

A.J.S. is funded by an NIHR Clinician Scientist Fellowship (NIHR-CS-011-028) and by the Medical Research Council, UK (MR/K015184/1).

## REFERENCES AND RECOMMENDED READING

Papers of particular interest, published within the annual period of review, have been highlighted as:

▪ of special interest
▪▪ of outstanding interest
